# Caffeine for the Pharmacological Treatment of Apnea of Prematurity in the NICU: Dose Selection Conundrum, Therapeutic Drug Monitoring and Genetic Factors

**DOI:** 10.3389/fphar.2021.681842

**Published:** 2021-07-26

**Authors:** Jia-Yi Long, Hong-Li Guo, Xin He, Ya-Hui Hu, Ying Xia, Rui Cheng, Xuan-Sheng Ding, Feng Chen, Jing Xu

**Affiliations:** ^1^ Pharmaceutical Sciences Research Center, Department of Pharmacy, Children’s Hospital of Nanjing Medical University, Nanjing, China; ^2^ School of Basic Medical Sciences and Clinical Pharmacy, China Pharmaceutical University, Nanjing, China; ^3^ Neonatal Intensive Care Unit, Children’s Hospital of Nanjing Medical University, Nanjing, China

**Keywords:** preterm infant, apnea of prematurity, caffeine, pharmacokinetics, therapeutic drug monitoring, polymorphism

## Abstract

Caffeine citrate is the drug of choice for the pharmacological treatment of apnea of prematurity. Factors such as maturity and genetic variation contribute to the interindividual variability in the clinical response to caffeine therapy in preterm infants, making the optimal dose administered controversial. Moreover, the necessity for therapeutic drug monitoring (TDM) of caffeine is still worth discussing due to the need to achieve the desired target concentrations as well as concerns about the safety of higher doses. Therefore, we reviewed the pharmacokinetic profile of caffeine in preterm infants, evidence of the safety and efficacy of different doses of caffeine, therapeutic concentration ranges of caffeine and impact of genetic variability on caffeine therapy. Whereas the safety and efficacy of standard-dose caffeine have been demonstrated, evidence for the safety of higher administered doses is insufficient. Thus, preterm infants who lack clinical response to standard-dose caffeine therapy are of interest for TDM when dose optimization is performed. Polymorphisms in pharmacodynamics-related genes, but not in pharmacokinetics-related genes, have a significant impact on the interindividual variability in clinical response to caffeine therapy. For preterm infants lacking clinical response, how to develop individualized medication regimens for caffeine remains to be explored.

## Introduction

Apnea of prematurity (AOP), classified as central, obstructive, or mixed, is usually defined as a cessation of breathing in a premature infant for 20 s or longer, or a shorter pause accompanied by bradycardia (<100 bpm), cyanosis, or pallor (Eichenwald, 2016). It is a common problem among preterm infants, particularly extremely preterm infants (Saroha and Patel, 2020). The reported incidence of AOP varies, but it is clearly inversely related to gestational age. Its incidence is 10% in neonates born beyond 34 weeks gestation. However, in newborns who are at 30–34 weeks gestation at birth, the incidence ranges from 20 to 85%. Ninety percent of the extremely low birth weight (less than 1,000 g) newborn population are reported to have AOP (Eichenwald, 2016; Erickson et al., 2021). Observational studies have demonstrated associations between apneic events and deficits in cerebral oxygenation (Schmid et al., 2015; Horne et al., 2017), increased risk for retinopathy of prematurity (ROP) (Di Fiore et al., 2010), neurodevelopmental impairment (Janvier et al., 2004; Martin et al., 2011), and even death or disability (Lodha et al., 2015).

Several interventions decrease apneic event frequency and duration. These include respiratory interventions including continuous positive airway pressure and pharmacologic therapies, such as methylxanthines, which have been used for over 40 years (Gentle et al., 2018). Caffeine is the first choice among all methylxanthines because of its efficacy, better tolerability and wider therapeutic index as well as longer half-life (Dobson and Hunt, 2013). Researchers of the international Caffeine for Apnea of Prematurity (CAP) trial confirmed the short- and long-term benefits and safety of neonatal caffeine therapy, including reduced rates of bronchopulmonary dysplasia (BPD), patent ductus arteriosus (PDA), and of severe ROP (Schmidt et al., 2006), and improved survival rates without neurodevelopmental disability at 18–21 months of age (Schmidt et al., 2007). Five- and 11 years follow-up studies confirmed that neonatal caffeine therapy appeared to have lasting beneficial effects on motor function and is effective and safe even into middle school age (Schmidt et al., 2012; Schmidt et al., 2017; Murner-Lavanchy et al., 2018). Therefore, caffeine has now become one of the most preferred drugs worldwide for AOP treatment and has been named the “silver” or “magic” bullet (Aranda et al., 2010; Bancalari, 2014).

Despite caffeine’s frequent use in routine neonatal practice, there are controversies surrounding this medicine, which future researches may resolve, including the optimal dose of caffeine administration (Moschino et al., 2020) and therapeutic drug monitoring (TDM) (Shrestha and Jawa, 2017; Saroha and Patel, 2020). Of note, neonatal caffeine therapy results in significant intersubject variability, and it remains unclear why apneic episodes persist in some preterm infants but not in others (He et al., 2020). Therefore, we summarize pharmacokinetic studies of caffeine in a population of preterm infants, as well as evidence of the safety, efficacy and therapeutic concentration ranges at different doses. We also discuss the dose optimization and the necessity for TDM of caffeine, and provide the first review of the impact of genetic variability on the clinical response to caffeine therapy.

## Pharmacokinetics of Caffeine in Preterm Infants

Most of the pharmacokinetic (PK) studies for caffeine were performed in premature neonates (Table 1 and Table 2). Due to the difficulty of adequate sampling in preterm babies, most studies have been population pharmacokinetic (PPK) studies using nonlinear mixed effects models (NONMEM) or P-pharm approaches (Table 2). The pharmacokinetics of caffeine is largely independent of the route of administration. Oral caffeine is almost completely bioavailable and is rapidly and completely absorbed from the gastrointestinal tract, reaching peak plasma concentrations in 30 min to 2 h after administration (Aranda et al., 1979a; Bonati et al., 1982; Blanchard and Sawers, 1983). Caffeine is hydrophilic and distributed evenly in all body fluids without tissue accumulation (Arnaud, 1976; Arnaud, 2011). It is also highly lipid-soluble to cross all biological membranes, including the blood-brain barrier, leading to a similar caffeine concentration between the plasma and cerebrospinal fluid of neonates (Turmen et al., 1979; Tanaka et al., 1984; Arnaud, 1987). The volume of distribution in preterm infants is mainly affected by the current body weight and gestational age, and its value is slightly greater than that in healthy adults, possibly due to the increased residence time of caffeine in the extracellular fluid (Aranda et al., 1979a; Bonati et al., 1982; Gorodischer and Karplus, 1982; Lelo et al., 1986; Thomson et al., 1996; Falcão et al., 1997; Lee et al., 1997; Lee et al., 2002; Kearns et al., 2003; Charles et al., 2008; Gao et al., 2020; Guo et al., 2020).

**TABLE 1:** Pharmacokinetics of caffeine in healthy adults and preterm infants.

First author, year^[ref]^	Number of cases	GA (weeks)	PNA (days)	BW (g)	CW (kg)	Route of administration	Dose of caffeine base (mg/kg)			T_max_ (minutes)	C_max_ (μg/ml)	CL (ml/kg/h)	V (L/kg)	t_1/2_
							S	L	M					
Healthy Adults														
Bonati et al. (1982)	4	NR	26–36^a^ ^,^ ^b^	NR	70	po	5.0			47	8.3	60.9	0.56	6.3
Lelo et al. (1986)	6	NR	19–21^a^ ^,^ ^b^	NR	62–104^a^	po	270^c^			NR	NR	124.2	0.63–0.71^a^	4.1
Preterm Infants														
Aranda et al. (1979a)	12	28.5	11.5	1,114.7	NR	iv	10.2	NR	11.2/day	30–120^a^	6–10	8.9	0.916	102.9
	3	30.0	19.7	1,334.3		po	10.0	NR	2.5/day					
	7	27.4	29.4	1,099.3		NR								
	10	27.7	35.2	1,041.5		NR								
Gorodischer and Karplus (1982)	13	30.6	1–42^a^	1,399	NR	iv	15 (1–7 doses)			NR	NR	8.5	0.781	65.0
Pearlman et al. (1989)	17	29.7	20.7	1,270	1.36	iv, po^d^		10	2.5–5 (1–2 doses/day)	NR	17.83	NR	NR	52.03
De Carolis et al. (1991)	5	30	0	1,670	NR	iv	5			NR	NR	NR	NR	72
	10	29.2	15	1,140		iv	5							
			NR			iv, po^e^		5	1.25/day					

Data are expressed as the mean, unless otherwise specified. NR, not reported; GA, gestational age; PNA, postnatal age; BW, birth weight; CW, current weight; T_max_, time to peak; C_max_, peak plasma concentration of caffeine; CL, clearance; V, volume of distribution; t_1/2_, elimination half-life; po, oral administration; iv, intravenous injection; S, single dose; L, loading dose; M, maintenance dose.

a Data are expressed as the range.

b Units are years.

c Unit is mg.

d 16 cases were administered orally and 1 case was administered intravenously.

e The loading dose was administered intravenously and the maintenance dose was administered orally.

**TABLE 2:** Population pharmacokinetics of caffeine in preterm infants.

First author, year^[ref]^	Number of cases	GA (weeks)	PNA (days)	BW (g)	CW (kg)	Dose of caffeine citrate		C_p_ (μg/ml)	CL (ml/kg/h)	V (L/kg)	t_1/2_	Modeling program	Pharmacokinetic parameters
						L (mg/kg)	M (mg/kg/day)						
Thomson et al. (1996)	80	25–41^a^	1–100^a^	600–2900^a^	NR	20	5	NR	7.9	0.64	NR	NONMEM	CL (L/day) = 0.14 × WT (kg) + 0.0024 × PNA (days)
													V (L) = 0.82
Lee et al. (1997)	38	28.2	4	1,167	NR	6	3	60.7	4.9	0.97	144	NONMEM	CL (L/h) = 0.00399 × CW (kg) + 0.000128 × PNA (days)
	39					30	15	31.1					V (L)^c^ = θ_1_ × CW (kg) + (θ_2_ × PNA (days)
	42					60	30	6.8					
Falcão et al. (1997)	75	23–35^a^	1–78^a^	600–2000^a^	NR	17.4–21.3^a^	2.1–9.5^a^	11.8	7.6	0.911	NR	NONMEM	CL (ml/h)^d^= (5.81 × CW [kg] + 1.22 × PNA [weeks]) × θ_1_ × θ_2_
													V (ml) = 911× CW (kg)
Lee et al. (2002)	18	28.9	NR	1,115.6	NR	20	5	3.6–28.4^a^	6.28	0.96	106	P-Pharm	CL (L/h) = 0.004248 × WT (kg) + 0.00154; r = 0.8, *p* < 0.01
													V (L) = 0.6299 × WT (kg) + 0.259; r = 0.67, *p* < 0.01
Charles et al. (2008)	59	27.6	12	1,009	0.992	80	20	47.4	7.0^b^	0.851^b^	101	NONMEM	CL (L/h) = 0.167 × (CW [kg]/70)^0.75^ × (PNA [days]/12)^0.358^
	51					20	5	14.7					V (L) = 58.7 × (CW [kg]/70)^0.75^
													K_a_ (h^−1^) = 1.48; F = 1.0
Guo et al. (2020)	46	28.97	21.22	1,240	1.39	20	8–10^a^	9.16–42.4^a^	10.2	2.494	NR	NONMEM	CL (L/h) = 0.268 × (CW [kg]/70)^0.75^
													V (L) = 109 × (CW [kg]/70) × e ^0.471×PNA (days)/19.5^
Gao et al. (2020)	99	28.51	24.87	1,129	1.306	20	5–10^a^	6.5–44.4^a^	12^b^	1.175^b^	NR	NONMEM	CL (L/h) = 0.0167 × (CW [g]/1,280)^0.75^ × (PMA [weeks]/31.1)^0.564^ × (CREA [μmol/L]/68)^−0.162^
													V (L) = 1.43 × (CW [g]/1,280)

Data are expressed as the mean, unless otherwise specified. NR, not reported; GA, gestational age; PNA, postnatal age; PMA, postmenstrual age; BW, birth weight; NONMEM, nonlinear mixed effects models; CW, current weight; WT, weight; L, loading dose; M, maintenance dose; C_P_, plasma concentration of caffeine; CL, clearance; V, volume of distribution; t_1/2_, elimination half-life; CREA, serum creatinine concentration.

a Data are expressed as the range.

b Data are expressed as the median.

c For GA > 28 weeks, θ_1_ = 0.764, θ_2_ = 0.0468; for GA ≤ 28 weeks, θ_1_ = 0.755, θ_2_ = 0.0224.

d If GA ≤ 28 weeks, θ_1_ = 0.757, otherwise = 1; if the current primary source of the patients’ nutrition is parenteral nutrition, θ_2_ = 0.836, otherwise = 1.

The metabolism of caffeine occurs primarily in the liver. In adults, with the catalysis by CYP2A1 and CYP2E1, caffeine undergoes 1-, 3-, and 7-demethylation to generate the biologically active metabolites theophylline, theobromine, and paraxanthine, which can then be further demethylated to monomethylxanthine (Gu et al., 1992; Thorn et al., 2012). Dimethylxanthine or monomethylxanthine is converted to methyluric acid by xanthine oxidase, whereas paraxanthine can also undergo 8-hydroxylation or generate 5-acetylamino-6-formylamino-3-methyluracil catalyzed by CYP2A6 or N-acetyltransferase-2, respectively (Begas et al., 2007; Thorn et al., 2012). However, in neonates, approximately 85% of caffeine is excreted unchanged in the urine, whereas this proportion in adults is less than 2% (Arnaud, 2011; Aldridge et al., 1979). CYP1A2 is the cytochrome P450 enzyme responsible for more than 90% of caffeine metabolism, studies have shown that CYP1A2 expression is not evident within the first 30 days of newborns’ life due to delayed ontogeny, and CYP1A2 content in liver microsomes of infants aged 1–3 months is only 10–15% of that in adults (Arnaud, 2011; Song et al., 2017; Sonnier and Cresteil, 1998). Correspondingly, the main metabolite in newborns during the first trimester of life is caffeine, whereas 8-hydroxylation appears early and matures approximately 1 month after birth, demethylation metabolism gradually matures with postnatal age, and acetylation is immature until at least 1 year of age (Aldridge et al., 1979; Carrier et al., 1988; Pons et al., 1989; Cazeneuve et al., 1994; al-Alaiyan et al., 2001; Blake et al., 2006). In addition, theophylline can be converted back to caffeine in premature infants by active methylation (Bory et al., 1978; Bory et al., 1979).

The serum half-life of caffeine in preterm infants is prolonged more than ten times that of adults because of immature hepatic metabolism and renal excretion (Aranda et al., 1979b; Bonati et al., 1982; Gorodischer and Karplus, 1982; Lelo et al., 1986; Pearlman et al., 1989; De Carolis et al., 1991). Caffeine’s clearance in preterm infants is influenced by various factors such as the current weight, postnatal age, gestational age, parenteral nutrition, and serum creatinine concentration, with values of approximately one-tenth of those in adults (Bonati et al., 1984; Lelo et al., 1986; Thomson et al., 1996; Falcão et al., 1997; Lee et al., 1997; Lee et al., 2002; Charles et al., 2008; Gao et al., 2020; Guo et al., 2020). For example, caffeine clearance shows a rapid maturation with postnatal age in a very recent study (Engbers et al., 2021). Earlier studies found that the elimination half-life and clearance of caffeine can reach adult levels at approximately 5–6 months after birth (Aranda et al., 1979b; Pons et al., 1988). However, a re-evaluation and validation of ontogeny functions for CYP1A2 describes an increase in relative intrisic metabolic clearance from birth to 3 years followed by a decrease to adult values (Salem et al., 2014). Therefore, the PK process of caffeine in neonates is variable and continues to mature with development, which needs to be taken into consideration when administered.

## Dosage of Caffeine in Preterm Infants

### Standard Dose of Caffeine and Its History

Caffeine is often available as caffeine citrate, which comes in both oral and injectable formulations, and the dose of caffeine base is half that of caffeine citrate (Shrestha and Jawa, 2017). As early as in 1977, Aranda et al. published the first study of caffeine used to treat AOP (Aranda et al., 1977). In that study, 18 preterm infants received an intravenous loading dose of 20 mg/kg caffeine citrate followed by a maintenance dose of 5–10 mg/kg once or twice daily for 2–3 days, and a marked reduction in apnea spells was observed. In the next 10 years, the same dose regimen was tested in several studies with small sample sizes (*n* = 16 to *n* = 23), and the therapeutic effect of caffeine on AOP was observed by comparison with placebo or theophylline (Murat et al., 1981; Brouard et al., 1985; Anwar et al., 1986; Bairam et al., 1987). In 1999, a multicenter, double-blind, randomized trial of caffeine citrate was performed using the above dose regimen. In this trial, eighty-five infants who were 28–32 weeks post-conception and 24 h or more after birth were randomized to caffeine or placebo for up to 10 days, and the results showed that this dose regimen was safe and effective for those recruited neonates (Erenberg et al., 2000). Based partly on such data, the U.S. Food and Drug Administration approved the dose regimen of caffeine citrate as a loading dose of 20 mg/kg followed by an intravenous or oral maintenance dose of 5 mg/kg/day, which is similar to what was approved by the European Medicines Agency (Erenberg et al., 2000; NDA 20-793/S-001, 2000; European Medicines Agency, 2009). Therefore, in this review, we refer to this as the “standard dose” regimen for caffeine.

In 2006, a large, multicenter, randomized, placebo-controlled trial, called the CAP trial, revealed the short- and long-term efficacy and safety of the standard dose regimen of caffeine (Schmidt et al., 2006; Schmidt et al., 2007). In the CAP trial, preterm infants with very low birth weight (VLBW, 500–1,250 g) were randomized to placebo or caffeine citrate at a loading dose of 20 mg/kg, followed by a maintenance dose of 5 mg/kg/24 h, which could be increased to 10 mg/kg/24 h for persistent apnea. This trial demonstrated several well-known beneficial short-terms effects of caffeine (Schmidt et al., 2006). Regarding the long-term effects, preterm infants had a higher rate of survival without neurodevelopmental disability and a lower incidence of severe ROP, cerebral palsy and cognitive delay at a corrected age of 18–21 months (Schmidt et al., 2007), with an improvement in gross motor function at 5 years (Schmidt et al., 2012). In addition, they also revealed that neonatal caffeine therapy at the doses used in CAP trial is effective and safe into miiddle school age (Doyle et al., 2017; Schmidt et al., 2017; Murner-Lavanchy et al., 2018; Schmidt et al., 2019). Due to the CAP trial, the standard-dose caffeine regimen has been widely used (Table 3). However, variable clinical outcomes do exist after standard-dose caffeine treatment.

**TABLE 3:** Main efficacy and safety results in standard dose caffeine treatment studies.

First author, year^[ref.]^	Study characteristics	Number of cases	Groups	Mean GA (weeks)	Mean PNA (days)	Dose of caffeine citrate		Main efficacy and safety results for caffeine treatment
						L (mg/kg)	M (mg/kg/day)	
Aranda et al. (1977)	noncontrolled	18	Caffeine	27.5	18.2			↓frequency of apnea (*p* < 0.001)
						20^a^	5–10 (2–3 days after L)	↓blood hydrogen ion concentration (*p* < 0.001)
								↓capillary carbon dioxide tension (*p* < 0.01) no significant change in heart rate
Murat et al. (1981)	randomized, controlled	18	Caffeine	30.1	13.2	20	5	↓apnea index^b^ on day 1 and 5 (*p* < 0.01)
			Control	29.8	16.1			↓apnea index^b^ from days 0–1 and from days 0–5 (*p* < 0.01) no adverse side effects
Brouard et al. (1985)	randomized	16	Caffeine	30.5	11.7	20	5	↓apnea frequency from days 0–1 (*p* < 0.001) and from days 0–5 (*p* < 0.001) in both groups no adverse effects
			Theophylline	30.5	11.6			
Anwar et al. (1986)	controlled	38^c^	Caffeine	32.0	35.0	20	5	↓apnea duration (*p* < 0.05)
			Control	32.2	39.9			↓percent periodic breathing (*p* < 0.05)
								↓apnea density (*p* < 0.05)
								4 infants were more irritable and restless
Bairam et al. (1987)	randomized, double-blind	20	Caffeine	30.3	6.2	20	2.5^d^	↑respiratory rates (*p* < 0.001) in both groups lower mean heart rate, smaller daily variations of mean plasma levels compared to theophylline group
			Theophylline	30.0	5.5			significant sodium loss
								no significant gastrointestinal side effects
Erenberg et al. (2000)	multicenter, randomized, double-blind, placebo-controlled	85	Caffeine	29.8	5.6	20	5	↓number of apnea episodes by ≥ 50% in 6 days (*p* < 0.05) eliminating apnea better in 5 days (*p* < 0.05)
			Placebo	29.9	4.9			no significant differences in number and percentage of adverse events
								caffeine citrate-related NEC in 1 infant
Schmidt et al. (2006)	multicenter, randomized, placebo-controlled (the CAP trial)	2,006	Caffeine	27	3^e^	20	5 (to 10 if apnea persisted)	↓duration of respiratory support (*p* < 0.01)
			Placebo	27	3^e^			↓cointerventions of doxapram, postnatal corticosteroids, and red-cell transfusions (*p* < 0.001)
								↓incidence of BPD (*p* < 0.001)
								↓PDA treatment (*p* < 0.001)
								↓weight gain temporarily (*p* < 0.05)
Schmidt et al. (2007)	follow-up reports of the CAP trial	1,869	Caffeine	18.8 months^e^ ^,^ ^f^				↓rate of death or disability (*p* = 0.008)
			Placebo	18.7 months^e^ ^,^ ^f^				↓incidence of cerebral palsy (*p* = 0.009)
								↓incidence of cognitive delay (*p* = 0.04)
								↓incidence of ROP > stage 3 (*p* = 0.01)
Schmidt et al. (2012)		1,640	Caffeine	5.2 years^e^ ^,^ ^f^				↑gross motor function (*p* = 0.006) no significant difference in death or disability (*p* = 0.09)
			Placebo	5.1 years^e^ ^,^ ^f^				
Schmidt et al. (2017)		920	Caffeine	11.4 years^e^ ^,^ ^f^				↓risk of motor impairment (*p* = 0.009) no significant differences in combined rate of academic, motor, and behavioral impairments (*p* = 0.07)
			Placebo	11.4 years^e^ ^,^ ^f^				
Murner-Lavanchy et al. (2018)		870	Caffeine	11.4 years^e^ ^,^ ^f^				↑motor coordination (*p* = 0.01)
			Placebo	11.4 years^e^ ^,^ ^f^				↑visuomotor integration (*p* < 0.05)
								↑visual perception (*p* = 0.02)
								↑visuospatial organization (*p* = 0.03) no significant differences in general intelligence, attention, executive function, and behavior
Doyle et al. (2017))		142	Caffeine	11.4 years^e^ ^,^ ^f^				↑expiratory flow rates in mid-childhood (*p* = 0.008)
			Placebo	11.4 years^e^ ^,^ ^f^				
Schmidt et al. (2019)		821	Caffeine	11.4 years^e^ ^,^ ^f^				↓social support and peer scores (50.8 vs. 52.6, *p* = 0.01) no significant differences in scores on other 9 dimensions of health-related quality of life
			Placebo	11.4 years^e^ ^,^ ^f^				

NR, not reported; GA, gestational age; PNA, postnatal age; L, loading dose; M, maintenance dose; NEC, necrotizing enterocolitis; BPD, bronchopulmonary dysplasia; PDA, patent ductus arteriosus; ROP, retinopathy of prematurity

a The initial dose was 20 mg/kg orally once or twice a day, and it was changed due to the accumulation of caffeine in the blood in preterm infants.

b Refers to the average number of apnea episodes per 100 min calculated from the recording within 24 h.

c The caffeine group additionally included four 14 day-old term infants with apnea.

d Dose regimen was 1.25 mg/kg every 12 h.

e Data are expressed as the median.

f Data are expressed as the corrected age.

### Higher Doses of Caffeine

Many studies have shown higher doses of caffeine to be more effective with negligible adverse effects (Table 4). Multiple studies have reported that higher doses of caffeine are more effective in reducing episodes of apnea and reducing extubation failure rates (Scanlon et al., 1992; Mohammed et al., 2015; Zhao et al., 2016; Wan et al., 2020). Among them, Mohammad et al. compared a higher dose (loading 40 mg/kg and maintenance of 20 mg/kg/day) with standard-dose caffeine citrate in 120 preterm infants < 32 weeks gestation with AOP within the first 10 days of life (Mohammed et al., 2015). In this trial, the higher dose of caffeine, in addition to being observed to have a better therapeutic effect, was also associated with a significant increase in tachycardia episodes. However, the clinical findings in this trial had no significant impact on physicians’ decision to withhold caffeine.

**TABLE 4:** Advantageous and Disadvantageous Results for Higher vs. Lower Doses of Caffeine in Randomized Controlled Trials.

First author, Year^[ref.]^	Type of study	Number of cases	GA, Other characteristics	Dose of caffeine citrate				Advantageous results for higher dose	Disadvantageous results for higher dose
				Higher dose		Lower dose			
				L	M	L	M		
Romagnoli et al. (1992)	Single center RCT	37^a^	<32	10	2.5	10	5		↑frequency of tachycardia and gastrointestinal intolerance (compared to other groups, *p* < 0.001)
Scanlon et al. (1992)	Single center RCT	44^b^	<31	50	12	25	6	↓more apnea episodes within 24 h (> 1/2 vs. 1/3)	
Steer et al. (2003)	Single center RCT	127	<32, ventilated for > 48 h	30	15	6	3	↓more documented apnea within 1 week after extubation (*p* = 0.01)	
				60	30				
				24 h before planned extubation, or within 6 h after unplanned extubation					
Steer et al. (2004)	Multicenter RCT	234	<30, ventilated for > 48 h	80	20	20	5	↓extubation failure (*p* < 0.01)	
				24 h before planned extubation, or within 6 h after unplanned extubation				↓duration of mechanical ventilation in infants GA < 28 weeks (*p* = 0.01)	
								↓documented apnea (*p* < 0.01)	
Gray et al. (2011)	Multicenter RCT	246	<30	80	20	20	5	↑mean general quotient (*p* = 0.048, after excluding two disabled children who could not be assessed, *p* = 0.075)	
Mohammed et al. (2015)	Single center RCT	120	<32	40	20	20	10	↓extubation failure (*p* = 0.02)	↑episodes of tachycardia (*p* = 0.04)
								↓frequency and days of documented apnea (*p* < 0.001)	
								↓duration of oxygen therapy (*p* = 0.04)	
McPherson et al. (2015)	Single center RCT	74	≤30	80 total over 36 h	10	30 total over 36 h	10		↑incidence of cerebellar hemorrhage (*p* = 0.03)
									↑hypertonicity (*p* = 0.02) and deviant neurologic signs (*p* = 0.04) at term equivalent age
Zhao et al. (2016)	Single center RCT	164	<32	20	15	20	5	↓frequency of apnea (*p* < 0.009)	
								↑success rate of removal of the ventilator (*p* = 0.015)	
								↑effective rate of caffeine treatment (*p* = 0.003)	
Zhang et al. (2019)	Single center RCT	78	28–32, born weight < 1,500 g	20	10	20	5	↑response rate of caffeine treatment (*p* = 0.035)	
								↓duration of apnea (*p* = 0.01) and time of caffeine treatment (*p* = 0.035)	
Wan et al. (2020)	Single center RCT	97	<30, ventilated for > 48 h	20	10	20	5	↓extubation failure (*p* = 0.017), age of extubation (*p* = 0.000), duration of invasive ventilation (*p* = 0.003), duration of ventilation before extubation (*p* = 0.000), and number of days of apnea (*p* = 0.001)	

GA, gestational age (weeks); L, loading dose (mg/kg); M, maintenance dose (mg/kg/day).

a A control group of 14 cases was included in the trial.

b An aminophylline group of 14 cases was included in the trial.

Other RCTs examined different dosing regimens of caffeine citrate for periextubation management of ventilated preterm infants. In 2003, Steer et al. compared three dose regimens of caffeine citrate (3, 15 and 30 mg/kg) for periextubation management of 127 preterm infants < 32 weeks gestation who were ventilated for > 48 h and found that there was no statistically significant difference in the incidence of extubation failure between different dosing groups (Steer et al., 2003). However, in a subsequent multicenter, double-blind RCT, the same authors found that a dose of 20 mg/kg was given 24 h before a planned extubation or within 6 h of an unplanned extubation reduced the rate of extubation failure within 48 h compared to a lower dose of 5 mg/kg, without evidence of harm in the first year of life (Steer et al., 2004). With the inclusion of additional subjects in the above-mentioned study, Gray compared the long-term effects of the two dose regimens used in Steer’s study (Gray et al., 2011). In this trial, 20 mg/kg/day caffeine citrate resulted in neither adverse outcomes in cognitive development, temperament, morbidity, mortality or disability at 1 year nor in behavior at 2 years.

Several findings also revealed the benefits of higher doses of caffeine for VLBW preterm infants. A retrospective analysis suggested that a higher average daily dose of caffeine citrate was associated with better neurodevelopmental outcomes in VLBW infants (Ravichandran et al., 2019). Another RCT trial found that a maintenance dose as high as 10 mg/kg better reduced the duration of apnea and caffeine treatment in this population (Zhang et al., 2019).

Four meta-analyses also synthesized the findings from the trials comparing higher and lower doses of caffeine citrate (Table 5). Among of them, three papers reported that higher caffeine dosage regimens might be better in reducing the risk of BPD and extubation failure (Chen et al., 2018; Pakvasa et al., 2018; Vliegenthart et al., 2018; Brattström et al., 2019), two reported a decrease in apnea frequency (Chen et al., 2018; Brattström et al., 2019) and one reported a shortened duration of mechanical ventilation (Brattström et al., 2019). Regarding safety concerns, three meta-analyses concluded a higher risk of tachycardia with higher dose of caffeine (Chen et al., 2018; Pakvasa et al., 2018), but no other adverse outcomes were increased.

**TABLE 5:** Results for Higher vs. Lower Doses of Caffeine in Meta-analyses.

First author, year^[ref.]^	Number of trials (patients)	Significant results (RR [95% CI]^a^, Number of patients)	Nonsignificant results (*p* > 0.05)
Vliegenthart et al. (2018)	6 RCTs (*n* = 620)	extubation failure (0.51 [0.37; 0.70], 463)	BPD, BPD combined mortality, hospital mortality, NEC ≥ grade 2, SIP, ROP ≥ grade 3, IVH > grade 2, hyperglycemia, mortality < 1year, major disability at 1 year, death or disability at 1 year, general quotient at 1 year
		tachycardia (3.39 [1.50; 7.64], 528)	
Brattström et al. (2019)	6 RCTs (*n* = 816)	BPD (0.76 [0.60; 0.96], 645) extubation failure (0.51 [0.36;0.71], 489)	hospital mortality, NEC, ROP ≥ grade 3, IVH ≥ grade 3, IVH, PVL, CBH, lesions indicative of brain injury, PDA treatment, major disabilities, seizure, somatic growth
		apnea frequency (−5.68 [−6.15; −5.22]^b^, 571)	
		tachycardia (2.56 [1.45; 4.50]^b^, 653)	
		MV duration (−1.69 [−2.13; −1.25]^b^, 727)	
Chen et al. (2018)	13 RCTs (*n* = 1,515)	BPD (0.79 [0.68; 0.91], 1,084) extubation failure (0.50 [0.35;0.71], 372)	hospital mortality, NEC, ROP, IVH, PVL, hyperglycemia, electrolyte disturbance, hypertension, feed intolerance, restlessness,
		apnea frequency (−1.55 [−2.72; −0.39]^b^, 168)	
		apnea duration (−4.85 [−8.29; −1.40]^b^, 150)	
		tachycardia (2.02 [1.30; 3.12], 880)	
Pakvasa et al. (2018)	3 RCTs (*n* = 432)	BPD (0.65 [0.65;0.97]^c^, 432)	

RCT, randomized controlled trial; BPD, bronchopulmonary dysplasia; MV, mechanical ventilation; NEC, necrotizing enterocolitis; SIP, spontaneous intestinal perforation; ROP, retinopathy of prematurity; IVH, intraventricular hemorrhage; PVL, periventricular leukomalacia; CBH, cerebellar hemorrhage; PDA, patent ductus arteriosus.

a Results are expressed as the relative ratio [95% confidence intervals], unless otherwise specified.

b Results are expressed as the mean differences [95% confidence intervals].

c Results are expressed as odds ratios [95% confidence intervals].

However, a pilot RCT found an increased incidence of cerebellar hemorrhage (CBH) in infants < 31 weeks’ gestation who were randomized to a higher-dose caffeine citrate (loading 80 mg/kg) (McPherson et al., 2015). Further analysis of this trial demonstrated that early high-dose caffeine therapy was associated with a trend toward an increase in seizure incidence (40 vs 58%, *p* = 0.1) and burden (48.9 vs 170.9, *p* = 0.1) (Vesoulis et al., 2016). These results discouraged a larger RCT. More recently, a retrospective study of 218 preterm infants < 28 weeks’ gestation who received a loading dose of caffeine citrate within the first 36 h of life was conducted (Firman et al., 2019). The use of early high loading dose caffeine citrate (a median dose of 80 mg/kg) was not shown to be associated with CBH. Although the two studies obtained different short-term outcomes, they both found that at 2 years of age, the Bayley-III scores used to assess neurodevelopment were not significantly different between the two dose groups.

Collectively, most previous RCTs had small sample sizes, and only two of them have reported 2 years clincal outcomes, which, although positive, need to be treated with caution. Thus, whether to use higher caffeine dosage regimens and how to optimize the caffeine dose are still questionable.

## Therapeutic Drug Monitoring of Caffeine

### Therapeutic Concentration of Standard Dose of Caffeine

The role of TDM for the control of therapeutic ranges of caffeine has often been challenged due to its benign safety profile when standard dosing is used. As early as in 1977, Aranda et al. revealed that the plasma concentration of standard dose caffeine needed to be monitored and the effective therapeutic concentration was established at 5–20 mg/L by referring to the use of theophylline (Aranda et al., 1977). Subsequently, the same authors also noted that the minimum effective plasma concentration of caffeine was 3–4 mg/L, but an optimal ventilatory response was observed at greater than 8 mg/L, and slight toxicity manifesting as temporary jitteriness was not detected until 50–84 mg/L (Aranda et al., 1979a; Aranda and Turmen, 1979). Therefore, Aranda et al. concluded that the optimal therapeutic concentration of caffeine is 8–20 mg/L, which both produces an adequate response to control apnea and avoids the risk of toxic effects (Aranda and Turmen, 1979).

Blood caffeine levels in preterm infants were almost within this conventional target range in other studies using similar standard dose regimens. In an RCT, 37 preterm infants rapidly achieved the therapeutic concentration within 24 h after starting treatment with a significant reduction in apneic episodes (Romagnoli et al., 1992). A study of 18 Asian preterm infants reported mean serum caffeine concentrations of 10–20 mg/L, and concluded that conventional caffeine therapeutic concentrations should be adhered to in order to ensure safety and efficacy (Lee et al., 2002). Leon et al. found that when the maintenance dose was 6 mg/kg, the 25th to 75th percentile range of mean serum caffeine concentrations in 108 preterm infants was comparable between two different loading dose groups (20 or 25 mg/kg), ranging from 18 to 23 mg/L (Leon et al., 2007). Another study found that the majority of preterm infants achieved target plasma caffeine levels of 5–20 mg/L when treated with a median dose of 5.0 mg/kg (range 2.5–10.9 mg/kg), with 95% of measures within this range in a cohort of 101 preterm infants with 23–32 weeks gestation, including those with renal or hepatic dysfunction (Natarajan et al., 2007a).

Therefore, blood caffeine concentrations of 5–20 or 8–20 mg/L have been commonly recognized as effective therapeutic concentrations for AOP treatment. Routine monitoring of caffeine levels is not recommended by the American Academy of Pediatrics Committee on Fetus and Newborn in their statement on AOP (Eichenwald, 2016). However, when we traced back to the origin, we recognized that the study by Aranda et al. was the fisrt study to determine the therapeutic concentration range of caffeine only based on 18 premature infants’ data (Aranda et al., 1977). Surprisingly, the blood caffeine concentrations were not measured in the well-known CAP trial and the drug was monitored according to its clinical effect only (Schmidt et al., 2006). Of note, the study by Natarajan et al. included a group of preterm neonates (*n* = 94) who lacked clinical response and had median to 75th quartile of plasma caffeine concentrations of 10.2–14.1 mg/L, suggesting that some neonates may need higher targets of caffeine to control apnea (Natarajan et al., 2007a). Collectively, whether to monitor the level of caffeine in preterm neonates using standard doses still needs to be explored.

### Therapeutic Concentration of Higher Dose of Caffeine

Many studies have shown that using higher dose of caffeine was more effective with negligible adverse effects than the standard-dose regimen and explored different effective therapeutic ranges of caffeine. A caffeine PK study including 13 premature infants found that the blood caffeine level varied widely from 12 to 36 mg/L when the single dose regimen of 15 mg/kg was used (Gorodischer and Karplus, 1982). Another RCT reported that 73% of the plasma caffeine concentration measurements in the high-dose group ranged from 26 to 40 mg/L, and apnea episodes were reduced more rapidly within 8 and 24 h without serious adverse effects compared to the standard-dose group (Scanlon et al., 1992). In a PK study conducted by Lee et al., in which no undesired consequences occurred when the mean serum caffeine concentrations were 35.8 or 69.0 mg/L (Lee et al., 1997), a therapeutic concentration > 35 mg/L was proposed to effectively prevent apnea after extubation. Similarly, Steer et al. reported that two higher dose groups with mean serum caffeine concentrations of 31.4 and 59.9 mg/L had short-term benefits and safety during peri-extubation among 127 infants < 32 weeks gestation (Steer et al., 2003). Subsequently, a commentary by Dr. Gal in 2007 questioned the traditional therapeutic concentration (Gal, 2007). According to his findings, higher serum caffeine concentrations produced more significant clinical responses including the reduced incidence of apnea, bradycardia, and of oxygen desaturation, which affirmed a target range of 8–40 mg/L, proposed by Natarajan et al. in another review (Natarajan et al., 2007b). In addition, a retrospective chart review of 198 infants born ≤ 29 weeks gestation showed that serum concentrations of caffeine > 14.5 mg/L were correlated with a reduction in the incidence of chronic lung disease (Chavez Valdez et al., 2011).

However, a small observational prospective study found that serum caffeine levels ≥ 20 mg/L were associated with increased proinflammatory cytokines in preterm infants during the first week of life (Alur et al., 2015). In another study of 115 preterm infants, there was no association between episodes of apnea and serum caffeine concentrations, although there was a significant but weak correlation between caffeine concentration and heart rate (Yu et al., 2016). Meanwhile, some cases reported acute intoxication due to overdose. A case report in 1980 presented two full-term infants with acute caffeine overdose who still had seizure activity when caffeine levels decreased to 31.9 mg/L and 10 mg/L, respectively, although the effect of perinatal asphyxia could not be ruled out (Banner and Czajka, 1980). Another 31 weeks gestational neonate experienced toxic reactions, including hypertonia, sweating, tachycardia, heart failure, pulmonary edema, metabolic disturbances and gastric dilatation, due to the blood caffeine level’s reaching 217.5 mg/L at 36.5 h after dosing, but these symptoms disappeared on day 7 at plasma concentrations of 60–70 mg/L (Anderson et al., 1999). Neurological symptoms, such as uninterrupted tremors, hypertonia, persistent reflex posture, crying, and digestive disorders were reported in a 33 weeks preterm newborn with a serum caffeine level of 160 mg/L at 66 h after administration, whereas his psychomotor development returned to normal after 3 months of age (Perrin et al., 1987). In addition, it is unfortunate that blood caffeine concentrations of subjects were not provided in most RCTs investigating doses of caffeine for AOP (Gray et al., 2011; McPherson et al., 2015; Mohammed et al., 2015; Zhao et al., 2016; Zhang et al., 2019; Wan et al., 2020). Due to lack of high-quality evidence for the long-term safety of high levels of caffeine, further determination of the therapeutic concentration range is difficult.

### Therapeutic Drug Monitoring and Dose Optimization of Caffeine

In the aforementioned studies, thetherapeutic concentration of caffeine was commonly recognized as 5–20 or 8–20 mg/L when using the standard dose regimen. However, some preterm neonates lacked a positive clinical response, although their caffeine levels were within the therapeutic concentration range, suggesting that these neonates may need to use higher doses to control apnea episodes. But using high doses may induce adverse reactions, and how to determine therapeutic doses for neonates who lack a clinical response still needs to be investigated. Therefore, is it feasible to guide dose optimization based on the monitoring caffeine levels?

Refer to *Therapeutic Concentration of Standard Dose of Caffeine*, routine monitoring of blood caffeine levels is generally not recommended. Leon et al. found that when a caffeine dose regimen close to standard (loading 20 or 25 mg/kg and maintenance of 6 mg/kg/day) was used, the serum drug concentrations were maintained in a safe therapeutic range and were independent of corrected gestational age, weight, and postnatal age within the first 2 weeks of life (Leon et al., 2007). Nevertheless, a PPK study found that the day-to-day variability in caffeine clearance of preterm neonates was twice the interindividual variability, implying that adjusting maintenance doses in light of previous serum concentrations is futile (Charles et al., 2008). However, some studies reported that higher levels of caffeine resulted in a greater response, and caffeine concentration monitoring was essential to ensure reaching the expected drug levels (Gal, 2007; Kahn and Godin, 2016). The 2019 guidelines of the National Institute for Health and Care Excellence recommend that caffeine levels should be monitored using reference ranges from the local laboratories to ensure safety when the daily maintenance dose is higher than 20 mg/kg (NICE, 2019). Combined with clinical practice, a growing body of research has endorsed the view that therapeutic monitoring of caffeine is of interest when therapeutic response is lacking or toxicity is suspected (Natarajan et al., 2007a; Gal, 2007; Leon et al., 2007; Gal, 2009; Kahn and Godin, 2016; Yu et al., 2016).

Naturally, the dose optimization of caffeine cannot be generalized. On the one hand, the change in caffeine clearance in preterm infants is a postnatal maturational progression (Aranda and Beharry, 2020). For routine use of caffeine, Koch et al. developed a simulated PK model and proposed an adjustment strategy based on postnatal age to maintain stable caffeine concentrations, with steps of increasing the caffeine maintenance daily dose by 1 mg/kg every 1 to 2 postnatal weeks, 6 mg/kg in the second week, 7 mg/kg in the third to fourth weeks, and 8 mg/kg in the fifth to eighth weeks (Koch et al., 2017). Recently, it has also been proposed that individualized caffeine medication can be administered with the help of a physiologic based pharmacokinetics (PBPK) model (Abduljalil et al., 2020; Aranda and Beharry, 2020; Verscheijden et al., 2020). On the other hand, the clinical response is specific to each individual and influenced by many factors such as gestational age, birth weight and genetic variability (Gal, 2007; Bloch-Salisbury et al., 2010; Francart et al., 2013; Ravichandran et al., 2019; He et al., 2020). Although increasing evidence has proven that the higher dose of caffeine is beneficial for newborns, there are also potential toxic risks and unknown long-term safety problems. In addition, the reported therapeutic concentration ranges of caffeine may not be simply combined together because of the differences such as the population, sample size, biological matrix, as well as assay methods in each study. This highlights the need to tailor the most appropriate range of individual therapeutic concentration according to blood caffeine levels, and the development of minimally invasive sampling techniques and noninvasive sampling of caffeine may contribute to achieving this requirement (Patel et al., 2013; Bruschettini et al., 2016; Chaabane et al., 2017).

## Impact of Genetic Variability on the Clinical Response to Caffeine Therapy

Earlier studies have found that heritability impacts the incidence of AOP, which raised interest in elucidating the effects of genetic factors on AOP as well as caffeine therapy (Tamim et al., 2003; Bloch-Salisbury et al., 2010). The therapeutic effect of caffeine depends on the disposition process of caffeine *in vivo*, that is, PK, and the interaction with target receptors, that is, pharmacodynamics (PD). Researches to date are precisely based on these two aspects.

In terms of PK, a recent retrospective study found that there were no significant differences in caffeine systemic exposure levels between apneic and apnea-free groups, as well as no significant association between the C_0_/D ratio and genetic variations in *CYP1A2* genes (rs2472299 and rs762551) (He et al., 2020). Correspondingly, in another PPK study of Chinese preterm neonates, the investigators found no significant association between several genetic variants in *CYP1A2* (rs2069514, rs2069521, rs2069526, rs2470890, rs35694136, rs3743484, rs56107638 and rs762551) and PK parameters (Gao et al., 2020). These findings echo delayed CYP1A2 ontogenesis and immature metabolism in premature infants, indicating that the contribution of genetic polymorphisms in caffeine-metabolizing enzymes to the variability in treatment response is limited. Notably, however, it has also been reported that the distribution of the aryl hydrocarbon receptor (*AHR*) CC genotype (rs4410790) differed significantly between the two groups with different responses to caffeine treatment in Chinese preterm neonates (He et al., 2020). Although AHR is normally a transcription factor that can regulate CYP1A2 expression, the authors stated that this finding may not be explained by the AHR-CYP1A2 metabolic pathway mechanisms.

In contrast, several studies have reported the effect of genetic polymorphisms associated with caffeine PD on treatment response. Adenosine receptor (AR) gene polymorphisms are the most described genetic factors in those current studies. AR is a class of G protein-coupled receptors with four known subtypes, A_1_, A_2A_, A_2B_ and A_3_, which is encoded by the *ADORA1*, *ADORA2A*, *ADORA2B*, and *ADORA3* genes, respectively (Chen et al., 2013; Borea et al., 2018). With a molecular structure similar to that of adenosine, caffeine acts as a nonspecific antagonist of A_1_AR and A_2A_AR to exert pharmacological effects at physiological concentrations (McLellan et al., 2016; Kumar and Lipshultz, 2019). In some studies, AR gene polymorphisms have already been found to be associated with intersubject variability in sensitivity to caffeine-induced anxiety (Childs et al., 2008; Rogers et al., 2010). Referring to these findings, Kumral et al. conducted a retrospective case-control study and found that *ADORA1* (rs16851030) CC genotype carriers had better responsiveness to caffeine than CT or TT genotype carriers.They also revealed that the correlation between *ADORA2A* (rs35320474, rs5751876, rs3761422) CT or TT genotypes and vulnerability to AOP as well as the correlation between *ADORA2A* (rs35320474) CT or TT genotypes and greater risk of BPD (Kumral et al., 2012). A significantly increased frequency of *ADORA2A* (rs5751876) CT, TT genotypes and T allele in caffeine nonresponders compared to caffeine responders was also reported in another prospective case-control study of Egyptian preterm neonates (Mokhtar et al., 2018). Moreover, a most recent retrospective study of Chinese preterm infants found that carriers of *ADORA1* T > G (rs10920568), G > T (rs12744240) and *ADORA3* C > A (rs10776727) as well as T > C (rs2298191) mutant genotypes did not respond to caffeine treatment, whereas *ADORA2A* T > A (rs34923252) and A > C (rs5996696) mutation genotype carriers responded better (He et al., 2020). In addition, this study also showed that a variant (rs521704, C > A) in the coding gene of adenosine dehydrogenase (ADA), which catalyzes adenosine metabolism, was associated with the response of premature infants to caffeine therapy (He et al., 2020). Phosphodiesterase (PDE), one of the targets of caffeine at nonphysiological concentrations, was also correlated, as carriers of the homozygous mutant genotype of *PDE4D* (rs10075508, C > T) responded poorly to standard-dose caffeine treatment (McLellan et al., 2016; Kumar and Lipshultz, 2019; He et al., 2020).

Collectively, although the sample size number of these studies is small, several genetic polymorphisms have been revealed to be associated with individual variances in response to caffeine therapy. Therefore, studies with larger sample sizes are needed to confirm these findings and further researches are warranted to explain how genetic variants play a critical role in the response to caffeine therapy in premature infants.

## Conclusion

Caffeine is effective in reducing apnea frequency in preterm neonates. The available evidence has confirmed the efficacy and safety of standard doses of caffeine, and routine TDM seems unnecessary in neonates who respond positively to caffeine treatment. However, the well-known CAP trial only started caffeine treatment when apnea occurred, and when to start standard-dose caffeine therapy is also a quite controversial issue that requires long-term safety studies. For developmental premature infants, a dosing adjustment strategy based on postnatal age was proposed to maintain stable caffeine concentrations, and individualized caffeine medication may be administered with the help of PPK and PBPK models. For neonates lacking a positive clinical response, as the evidence for the use of higher doses of caffeine is insufficient, and TDM should be performed to achieve the desired blood caffeine level and ensure safety. The long-term results of larger trials of higher doses of caffeine are expected and would be more reasonable if corresponding blood caffeine concentrations could be provided. In addition, the study of genetic factors has preliminarily revealed the association between genetic polymorphisms and clinical response to caffeine therapy. Further studies are required to explain how genetic variants play a role in the response to caffeine therapy in premature infants. And how to establish an approach to individualize medication regimens for infants with poor clinical response by integrating tools such as TDM, genetic testing, PPK and PBPK models is also a direction for future exploration.

## Abbreviations

ADA, adenosine dehydrogenase; AHR, aryl hydrocarbon receptor; AOP, apnea of prematurity; AR, adenosine receptor; BPD, bronchopulmonary dysplasia; CAP, Caffeine for Apnea of Prematurity; CBH, cerebellar hemorrhage; NONMEM, nonlinear mixed effects models; PBPK, physiologic based pharmacokinetics; PD, pharmacodynamics; PDA, patent ductus arteriosus; PDE, phosphodiesterase; PK, pharmacokinetics; PPK, population pharmacokinetics; RCT, randomized controlled trial; ROP, retinopathy of prematurity; TDM, therapeutic drug monitoring; VLBW, very low birth weight.
